# Ethyl 4-(3-hydroxy­phen­yl)-2,7,7-trimethyl-5-oxo-1,4,5,6,7,8-hexa­hydro­quinoline-3-carboxyl­ate

**DOI:** 10.1107/S1600536809039877

**Published:** 2009-10-07

**Authors:** P. Mookiah, K. Rajesh, T. Narasimhamurthy, V. Vijayakumar, N. Srinivasan

**Affiliations:** a Department of Physics, Thiagarajar College, Madurai 625 009, India; b Organic Chemistry Division, School of Science, VIT University, Vellore 632 014, India; c Materials Research Centre, Indian Institute of Science, Bangalore 560 012, India; d Organic Chemistry Division, School of Science, VIT University, Vellore 632 014, India

## Abstract

In the mol­ecular structure of the title compound, C_21_H_25_NO_4_, the dihydro­pyridine ring adopts a flattened boat conformation while the cyclo­hexenone ring is in an envelope conformation. In the crystal structure, mol­ecules are linked into a two-dimensional network parallel to (10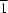) by N—H⋯O and O—H⋯O hydrogen bonds. The network is generated by *R*_4_^4^(30) and *R*_4_^4^(34) graph-set motifs.

## Related literature

For general background to oxoquinoline derivatives, see: Baba (1997 ▶); Baba *et al.* (1997 ▶,1998 ▶); Koga *et al.* (1980 ▶); Qi *et al.* (2007 ▶). For a related structure, see: Czaun *et al.* (2002 ▶); For graph-set motifs, see: Etter *et al.* (1990 ▶).
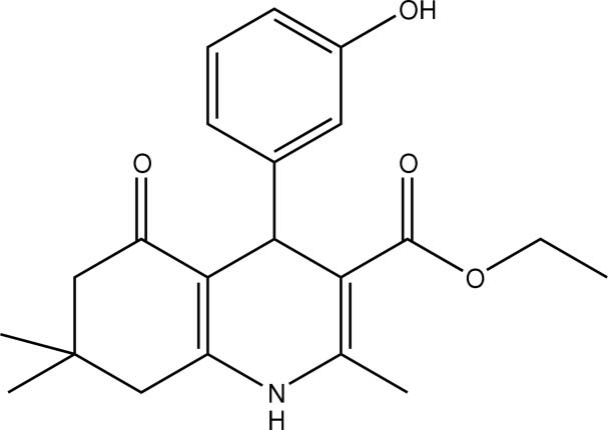


## Experimental

### 

#### Crystal data

C_21_H_25_NO_4_
*M* *_r_* = 355.42
Monoclinic, 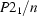

*a* = 10.8721 (4) Å
*b* = 16.1255 (7) Å
*c* = 11.0856 (4) Å
β = 100.682 (2)°
*V* = 1909.83 (13) Å^3^
*Z* = 4
Mo *K*α radiation
μ = 0.09 mm^−1^
*T* = 296 K
0.26 × 0.15 × 0.12 mm

#### Data collection

Bruker Kappa APEXII area-detector diffractometer
Absorption correction: multi-scan (*SADABS*; Sheldrick, 2004 ▶) *T*_min_ = 0.93, *T*_max_ = 0.95
14667 measured reflections
3163 independent reflections
2137 reflections with *I* > 2σ(*I*)
*R*_int_ = 0.041

#### Refinement

*R*[*F* ^2^ > 2σ(*F* ^2^)] = 0.040
*wR*(*F* ^2^) = 0.115
*S* = 1.02
3163 reflections
236 parameters
H-atom parameters constrained
Δρ_max_ = 0.15 e Å^−3^
Δρ_min_ = −0.15 e Å^−3^

### 

Data collection: *APEX2* (Bruker, 2004 ▶); cell refinement: *SAINT-Plus* (Bruker, 2004 ▶); data reduction: *SAINT-Plus*; program(s) used to solve structure: *SHELXS97* (Sheldrick, 2008 ▶); program(s) used to refine structure: *SHELXL97* (Sheldrick, 2008 ▶); molecular graphics: *PLATON* (Spek, 2009 ▶); software used to prepare material for publication: *SHELXL97*.

## Supplementary Material

Crystal structure: contains datablocks I, global. DOI: 10.1107/S1600536809039877/ci2916sup1.cif

Structure factors: contains datablocks I. DOI: 10.1107/S1600536809039877/ci2916Isup2.hkl

Additional supplementary materials:  crystallographic information; 3D view; checkCIF report

## Figures and Tables

**Table 1:** Hydrogen-bond geometry (Å, °)

*D*—H⋯*A*	*D*—H	H⋯*A*	*D*⋯*A*	*D*—H⋯*A*
O8*C*—H8*C*⋯O9*B*^i^	0.82	2.05	2.835 (2)	162
N1—H1⋯O6*A*^ii^	0.86	2.16	2.970 (2)	157

Symmetry codes: (i) 
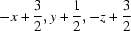
; (ii) 
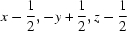
.

**Fig. 1.**
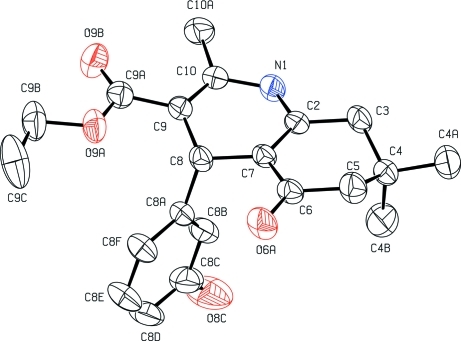
The molecular structure of the title compound, with atom labels and 50% probability displacement ellipsoids for non-H atoms. H atoms have been omitted for clarity.

**Fig. 2.**
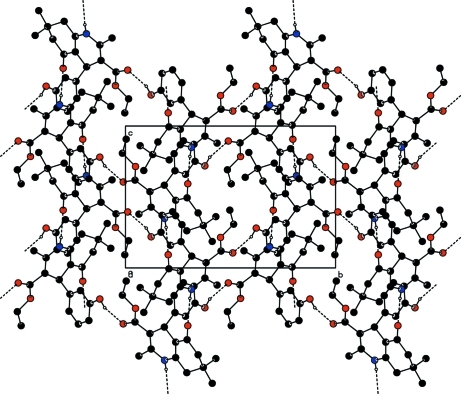
A view of the molecular aggregation down the a axis. Hydrogen bonds are shown as dashed lines. C-bound H atoms have been omitted for clarity.

**Fig. 3.**
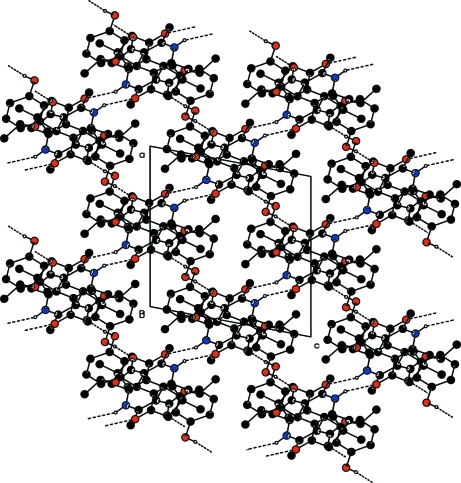
A view of the molecular aggregation down the b axis. Hydrogen bonds are shown as dashed lines. C-bound H atoms have been omitted for clarity.

## supplementary crystallographic information

### Comment

Some oxoquinoline derivatives *viz*. 8-difluoromethoxy-1-ethyl-6-fluoro-1,4-dihydro-7-[4-(2-methoxyphenyl)- 1-πiperazinyl]- 4-oxoquinoline-3-carboxylic acid (K-12), 7-(3,4-dehydro-4-phenyl-1-piperidinyl)-1,4-dihydro-6-fluoro-1-methyl- 8-trifluoromethyl-4-oxoquinoline-3-carboxylic acid (K-37), 8-difluoromethoxy-1,4-dihydro-6-fluoro-7-(3,4-dehydro-4-phenyl- 1-piperidinyl)-1-[4,(1,2,4-triazol-1-yl)methylphenyl]-4-oxoquinoline- 3-carboxylic acid (K-38) act as potent and selective inhibitor of human immunodeficiency virus type I (HIV-1) transcription (Baba, 1997; Baba *et al.*, 1997,1998). Structure-activity relationships of antibacterial oxoquinolone-3-carboxylic acids have been studied (Koga *et al.*, 1980). In view of the signficicant biological activitiy, precise single crystal structure determinations of these derivatives are expcted to provide insights in their design and function. The crystal structure of 1*H*-2-phenyl-3-hydroxy-4-oxoquinoline-dimethylsulfoxide has already been reported (Czaun *et al.*, 2002). The expression, purification and crystallization of 1*H*-3-hydroxy-4-oxoquinoline 2,4-dioxygenase are reported elsewhere (Qi *et al.*, 2007).

The dihydropyridine ring of the title molecule (Fig.1) adopts a flattened boat conformation. The cyclohexenone ring is in an envelope conformation with atom C4 at the flap. The 4-methoxyphenyl ring and the planar part of the dihydropyridine ring (C2/C7/C9/C10) are nearly perpendicular to each other, with a dihedral angle of 89.37 (6)°.

In the crystal structure, molecules are linked into a two-dimensional network (Fig.2) parallel to the (101) by N—H···O and O—H···O hydrogen bonds (Table 1). The two-deimensional layer, resembiling a corrugated sheet, contains *R*_4_^4^(30) and *R*_4_^4^(34) graph-set motifs (Etter *et al.*, 1990) as its fundamental repeating units. It is observed that these rings are assembled through centrosymmetrically related pairs of molecules with no direct hydrogen bonding between them.

### Experimental

A 50 ml round-bottomed flask was charged with 3-hydroxybenzaldehyde (1.221 g, 10 mmol), 5,5-dimethyl-1,3-cyclohexanedione (1.402 g, 10 mmol), ethyl acetoacetate (1.265 ml, 10 mmol) and ammonium acetate (0.771 g, 10 mmol) followed by ethanol (10 ml). The mixture was stirred at 343 K for 1.5 h and left aside for a day. The solid separated out was filtered and washed with ethanol-diethyl ether mixture (1:4). It was recrystalyzed from 100% chloroform. Light yellow prismatic crystals of the title compound were obtained by slow evaporation of a methonolic solution. Pale yellow crystals with slab morphology were obtained by slow evaporation of a methonol-chloroform solution.

### Refinement

H atoms were positioned geometrically [O-H = 0.82 Å, N-H = 0.86 Å and C-H = 0.93–0.98 Å] and refined using a riding model with *U*_iso_(H) = 1.2*U*_eq_(C) and 1.2*U*_eq_(O and C_methyl_).

### Crystal data

**Table:**

C_21_H_25_NO_4_	*F*(000) = 760
*M**_r_* = 355.42	*D*_x_ = 1.236 Mg m^−^^3^
Monoclinic, *P*2_1_/*n*	Mo *K*α radiation, λ = 0.71073 Å
Hall symbol: -P 2yn	Cell parameters from 5123 reflections
*a* = 10.8721 (4) Å	θ = 2.0–30.0°
*b* = 16.1255 (7) Å	µ = 0.09 mm^−^^1^
*c* = 11.0856 (4) Å	*T* = 296 K
β = 100.682 (2)°	Prism, yellow
*V* = 1909.83 (13) Å^3^	0.26 × 0.15 × 0.12 mm
*Z* = 4	

### Data collection

**Table:**

Bruker Kappa APEXII area-detector diffractometer	3163 independent reflections
Radiation source: fine-focus sealed tube	2137 reflections with *I* > 2σ(*I*)
graphite	*R*_int_ = 0.041
ω and φ scans	θ_max_ = 24.5°, θ_min_ = 2.3°
Absorption correction: multi-scan (*SADABS*; Sheldrick, 2004)	*h* = −12→12
*T*_min_ = 0.93, *T*_max_ = 0.95	*k* = −18→17
14667 measured reflections	*l* = −11→12

### Refinement

**Table:**

Refinement on *F*^2^	Primary atom site location: structure-invariant direct methods
Least-squares matrix: full	Secondary atom site location: difference Fourier map
*R*[*F*^2^ > 2σ(*F*^2^)] = 0.040	Hydrogen site location: inferred from neighbouring sites
*wR*(*F*^2^) = 0.115	H-atom parameters constrained
*S* = 1.02	*w* = 1/[σ^2^(*F*_o_^2^) + (0.048*P*)^2^ + 0.4479*P*] where *P* = (*F*_o_^2^ + 2*F*_c_^2^)/3
3163 reflections	(Δ/σ)_max_ = 0.001
236 parameters	Δρ_max_ = 0.15 e Å^−^^3^
0 restraints	Δρ_min_ = −0.15 e Å^−^^3^

### Special details

**Table:**

Geometry. All e.s.d.'s (except the e.s.d. in the dihedral angle between two l.s. planes) are estimated using the full covariance matrix. The cell e.s.d.'s are taken into account individually in the estimation of e.s.d.'s in distances, angles and torsion angles; correlations between e.s.d.'s in cell parameters are only used when they are defined by crystal symmetry. An approximate (isotropic) treatment of cell e.s.d.'s is used for estimating e.s.d.'s involving l.s. planes.
Refinement. Refinement of *F*^2^ against ALL reflections. The weighted *R*-factor *wR* and goodness of fit *S* are based on *F*^2^, conventional *R*-factors *R* are based on *F*, with *F* set to zero for negative *F*^2^. The threshold expression of *F*^2^ > σ(*F*^2^) is used only for calculating *R*-factors(gt) *etc*. and is not relevant to the choice of reflections for refinement. *R*-factors based on *F*^2^ are statistically about twice as large as those based on *F*, and *R*- factors based on ALL data will be even larger.

### Fractional atomic coordinates and isotropic or equivalent isotropic displacement parameters (Å^2^)

**Table:**

	*x*	*y*	*z*	*U*_iso_*/*U*_eq_	
O9A	1.01275 (14)	0.04777 (9)	0.71484 (12)	0.0559 (4)	
O6A	1.21908 (13)	0.29658 (10)	0.59297 (11)	0.0577 (4)	
O9B	0.84079 (15)	−0.01070 (9)	0.60571 (13)	0.0618 (4)	
O8C	0.72595 (19)	0.37790 (12)	0.71929 (17)	0.0969 (7)	
H8C	0.7098	0.4009	0.7804	0.145*	
N1	0.85621 (15)	0.18610 (10)	0.34959 (13)	0.0444 (4)	
H1	0.7990	0.1933	0.2857	0.053*	
C7	1.03735 (16)	0.23880 (11)	0.47694 (14)	0.0346 (4)	
C2	0.95697 (17)	0.23874 (11)	0.36798 (15)	0.0365 (4)	
C8	1.01637 (17)	0.18488 (12)	0.58296 (15)	0.0376 (5)	
H8	1.0962	0.1583	0.6176	0.045*	
C9	0.92188 (17)	0.11633 (11)	0.53752 (15)	0.0368 (4)	
C6	1.14473 (17)	0.29327 (12)	0.49410 (15)	0.0394 (5)	
C10	0.84203 (17)	0.12208 (12)	0.42851 (15)	0.0384 (5)	
C9A	0.91749 (19)	0.04511 (13)	0.61867 (17)	0.0433 (5)	
C4	1.04698 (18)	0.37054 (12)	0.29899 (16)	0.0435 (5)	
C8B	0.8704 (2)	0.28573 (13)	0.65967 (17)	0.0509 (6)	
H8B	0.8260	0.2893	0.5796	0.061*	
C3	0.97318 (19)	0.29188 (13)	0.26167 (15)	0.0471 (5)	
H3A	0.8913	0.3068	0.2159	0.057*	
H3B	1.0157	0.2600	0.2075	0.057*	
C5	1.16617 (19)	0.34564 (14)	0.38772 (17)	0.0537 (6)	
H5A	1.2199	0.3154	0.3424	0.064*	
H5B	1.2103	0.3956	0.4196	0.064*	
C8A	0.97467 (19)	0.23549 (12)	0.68484 (15)	0.0421 (5)	
C10A	0.7372 (2)	0.06394 (14)	0.37939 (18)	0.0536 (6)	
H10A	0.7707	0.0101	0.3675	0.080*	
H10B	0.6924	0.0845	0.3024	0.080*	
H10C	0.6813	0.0600	0.4368	0.080*	
C8F	1.0398 (2)	0.23138 (15)	0.80510 (17)	0.0608 (6)	
H8F	1.1111	0.1986	0.8241	0.073*	
C8C	0.8307 (2)	0.33082 (14)	0.7515 (2)	0.0608 (6)	
C9B	1.0157 (2)	−0.01484 (16)	0.8081 (2)	0.0682 (7)	
H91B	1.0250	−0.0694	0.7740	0.082*	
H92B	0.9384	−0.0139	0.8400	0.082*	
C4A	1.0813 (2)	0.41285 (15)	0.18658 (18)	0.0673 (7)	
H41A	1.1279	0.4625	0.2115	0.101*	
H42A	1.0063	0.4268	0.1298	0.101*	
H43A	1.1313	0.3759	0.1477	0.101*	
C8E	0.9988 (3)	0.27565 (18)	0.8960 (2)	0.0777 (8)	
H8E	1.0422	0.2714	0.9763	0.093*	
C8D	0.8956 (3)	0.32578 (17)	0.8710 (2)	0.0717 (8)	
H8D	0.8697	0.3559	0.9333	0.086*	
C4B	0.9704 (3)	0.43009 (15)	0.3613 (2)	0.0796 (8)	
H41B	1.0181	0.4796	0.3844	0.119*	
H42B	0.9502	0.4043	0.4332	0.119*	
H43B	0.8947	0.4440	0.3056	0.119*	
C9C	1.1234 (3)	0.0034 (2)	0.9074 (2)	0.0993 (11)	
H91C	1.1276	−0.0374	0.9710	0.149*	
H92C	1.1130	0.0574	0.9407	0.149*	
H93C	1.1993	0.0022	0.8749	0.149*	

### Atomic displacement parameters (Å^2^)

**Table:**

	*U*^11^	*U*^22^	*U*^33^	*U*^12^	*U*^13^	*U*^23^
O9A	0.0679 (10)	0.0550 (9)	0.0430 (7)	0.0030 (7)	0.0057 (7)	0.0178 (7)
O6A	0.0501 (9)	0.0749 (11)	0.0390 (7)	−0.0106 (8)	−0.0157 (7)	0.0038 (7)
O9B	0.0681 (11)	0.0521 (10)	0.0667 (10)	−0.0076 (8)	0.0164 (8)	0.0124 (8)
O8C	0.1049 (15)	0.1028 (16)	0.0858 (13)	0.0344 (13)	0.0246 (11)	−0.0337 (11)
N1	0.0460 (10)	0.0493 (10)	0.0313 (8)	−0.0099 (8)	−0.0099 (7)	0.0028 (8)
C7	0.0365 (10)	0.0380 (11)	0.0272 (9)	0.0017 (8)	0.0006 (7)	−0.0014 (8)
C2	0.0402 (11)	0.0380 (11)	0.0286 (9)	−0.0024 (9)	−0.0005 (8)	−0.0028 (8)
C8	0.0390 (11)	0.0427 (11)	0.0282 (9)	0.0027 (9)	−0.0011 (8)	0.0036 (8)
C9	0.0438 (11)	0.0356 (11)	0.0325 (9)	0.0021 (9)	0.0107 (8)	−0.0012 (8)
C6	0.0375 (11)	0.0456 (12)	0.0312 (9)	0.0008 (9)	−0.0034 (8)	−0.0018 (9)
C10	0.0438 (11)	0.0374 (11)	0.0338 (9)	−0.0024 (9)	0.0068 (8)	−0.0046 (9)
C9A	0.0479 (12)	0.0432 (12)	0.0418 (11)	0.0067 (10)	0.0160 (10)	0.0002 (9)
C4	0.0519 (12)	0.0440 (12)	0.0312 (9)	−0.0051 (10)	−0.0009 (9)	0.0020 (9)
C8B	0.0619 (14)	0.0548 (14)	0.0351 (10)	0.0008 (11)	0.0063 (10)	−0.0091 (10)
C3	0.0542 (13)	0.0540 (13)	0.0282 (9)	−0.0119 (10)	−0.0052 (9)	0.0032 (9)
C5	0.0508 (13)	0.0627 (14)	0.0429 (11)	−0.0151 (11)	−0.0037 (10)	0.0060 (10)
C8A	0.0538 (13)	0.0429 (12)	0.0285 (9)	−0.0071 (10)	0.0046 (8)	−0.0019 (8)
C10A	0.0579 (14)	0.0541 (14)	0.0472 (11)	−0.0153 (11)	0.0058 (10)	−0.0075 (10)
C8F	0.0772 (16)	0.0680 (15)	0.0326 (11)	−0.0053 (13)	−0.0016 (10)	−0.0034 (11)
C8C	0.0738 (16)	0.0540 (15)	0.0586 (14)	−0.0031 (13)	0.0229 (12)	−0.0155 (12)
C9B	0.0835 (18)	0.0674 (16)	0.0571 (13)	0.0206 (13)	0.0220 (13)	0.0296 (12)
C4A	0.0850 (18)	0.0689 (16)	0.0438 (12)	−0.0258 (14)	0.0008 (11)	0.0110 (11)
C8E	0.109 (2)	0.089 (2)	0.0303 (11)	−0.0111 (18)	0.0015 (13)	−0.0135 (12)
C8D	0.101 (2)	0.0724 (18)	0.0468 (13)	−0.0194 (16)	0.0261 (14)	−0.0260 (13)
C4B	0.117 (2)	0.0556 (16)	0.0675 (15)	0.0266 (15)	0.0204 (15)	0.0067 (13)
C9C	0.0805 (19)	0.148 (3)	0.0679 (16)	0.0255 (19)	0.0092 (15)	0.0560 (19)

### Geometric parameters (Å, °)

**Table:**

O9A—C9A	1.342 (2)	C3—H3A	0.97
O9A—C9B	1.442 (2)	C3—H3B	0.97
O6A—C6	1.237 (2)	C5—H5A	0.97
O9B—C9A	1.217 (2)	C5—H5B	0.97
O8C—C8C	1.361 (3)	C8A—C8F	1.390 (3)
O8C—H8C	0.82	C10A—H10A	0.96
N1—C2	1.371 (2)	C10A—H10B	0.96
N1—C10	1.380 (2)	C10A—H10C	0.96
N1—H1	0.86	C8F—C8E	1.374 (3)
C7—C2	1.353 (2)	C8F—H8F	0.93
C7—C6	1.445 (3)	C8C—C8D	1.383 (3)
C7—C8	1.513 (2)	C9B—C9C	1.480 (3)
C2—C3	1.494 (3)	C9B—H91B	0.97
C8—C9	1.529 (3)	C9B—H92B	0.97
C8—C8A	1.529 (3)	C4A—H41A	0.96
C8—H8	0.98	C4A—H42A	0.96
C9—C10	1.354 (2)	C4A—H43A	0.96
C9—C9A	1.465 (3)	C8E—C8D	1.369 (4)
C6—C5	1.503 (3)	C8E—H8E	0.93
C10—C10A	1.498 (3)	C8D—H8D	0.93
C4—C3	1.517 (3)	C4B—H41B	0.96
C4—C4B	1.518 (3)	C4B—H42B	0.96
C4—C4A	1.526 (3)	C4B—H43B	0.96
C4—C5	1.528 (3)	C9C—H91C	0.96
C8B—C8A	1.379 (3)	C9C—H92C	0.96
C8B—C8C	1.383 (3)	C9C—H93C	0.96
C8B—H8B	0.93		
			
C9A—O9A—C9B	117.31 (17)	C4—C5—H5B	108.6
C8C—O8C—H8C	109.5	H5A—C5—H5B	107.6
C2—N1—C10	123.30 (14)	C8B—C8A—C8F	118.38 (19)
C2—N1—H1	118.4	C8B—C8A—C8	120.62 (15)
C10—N1—H1	118.4	C8F—C8A—C8	120.99 (19)
C2—C7—C6	119.35 (16)	C10—C10A—H10A	109.5
C2—C7—C8	121.84 (17)	C10—C10A—H10B	109.5
C6—C7—C8	118.79 (14)	H10A—C10A—H10B	109.5
C7—C2—N1	119.95 (17)	C10—C10A—H10C	109.5
C7—C2—C3	123.56 (17)	H10A—C10A—H10C	109.5
N1—C2—C3	116.47 (14)	H10B—C10A—H10C	109.5
C7—C8—C9	110.38 (14)	C8E—C8F—C8A	120.1 (2)
C7—C8—C8A	112.04 (15)	C8E—C8F—H8F	120.0
C9—C8—C8A	110.86 (15)	C8A—C8F—H8F	120.0
C7—C8—H8	107.8	O8C—C8C—C8B	117.4 (2)
C9—C8—H8	107.8	O8C—C8C—C8D	122.5 (2)
C8A—C8—H8	107.8	C8B—C8C—C8D	120.1 (2)
C10—C9—C9A	120.87 (17)	O9A—C9B—C9C	107.7 (2)
C10—C9—C8	121.66 (16)	O9A—C9B—H91B	110.2
C9A—C9—C8	117.43 (15)	C9C—C9B—H91B	110.2
O6A—C6—C7	121.49 (17)	O9A—C9B—H92B	110.2
O6A—C6—C5	120.01 (17)	C9C—C9B—H92B	110.2
C7—C6—C5	118.50 (14)	H91B—C9B—H92B	108.5
C9—C10—N1	119.23 (16)	C4—C4A—H41A	109.5
C9—C10—C10A	126.89 (18)	C4—C4A—H42A	109.5
N1—C10—C10A	113.86 (15)	H41A—C4A—H42A	109.5
O9B—C9A—O9A	121.89 (18)	C4—C4A—H43A	109.5
O9B—C9A—C9	127.35 (18)	H41A—C4A—H43A	109.5
O9A—C9A—C9	110.76 (17)	H42A—C4A—H43A	109.5
C3—C4—C4B	110.26 (19)	C8D—C8E—C8F	121.6 (2)
C3—C4—C4A	110.37 (15)	C8D—C8E—H8E	119.2
C4B—C4—C4A	109.04 (18)	C8F—C8E—H8E	119.2
C3—C4—C5	107.36 (16)	C8E—C8D—C8C	118.8 (2)
C4B—C4—C5	110.14 (17)	C8E—C8D—H8D	120.6
C4A—C4—C5	109.66 (17)	C8C—C8D—H8D	120.6
C8A—C8B—C8C	121.09 (19)	C4—C4B—H41B	109.5
C8A—C8B—H8B	119.5	C4—C4B—H42B	109.5
C8C—C8B—H8B	119.5	H41B—C4B—H42B	109.5
C2—C3—C4	113.47 (14)	C4—C4B—H43B	109.5
C2—C3—H3A	108.9	H41B—C4B—H43B	109.5
C4—C3—H3A	108.9	H42B—C4B—H43B	109.5
C2—C3—H3B	108.9	C9B—C9C—H91C	109.5
C4—C3—H3B	108.9	C9B—C9C—H92C	109.5
H3A—C3—H3B	107.7	H91C—C9C—H92C	109.5
C6—C5—C4	114.60 (16)	C9B—C9C—H93C	109.5
C6—C5—H5A	108.6	H91C—C9C—H93C	109.5
C4—C5—H5A	108.6	H92C—C9C—H93C	109.5
C6—C5—H5B	108.6		
			
C6—C7—C2—N1	178.61 (17)	C10—C9—C9A—O9A	173.47 (17)
C8—C7—C2—N1	−3.0 (3)	C8—C9—C9A—O9A	−8.8 (2)
C6—C7—C2—C3	0.7 (3)	C7—C2—C3—C4	−26.3 (3)
C8—C7—C2—C3	179.07 (17)	N1—C2—C3—C4	155.73 (17)
C10—N1—C2—C7	−11.5 (3)	C4B—C4—C3—C2	−70.4 (2)
C10—N1—C2—C3	166.61 (17)	C4A—C4—C3—C2	169.08 (18)
C2—C7—C8—C9	17.0 (2)	C5—C4—C3—C2	49.6 (2)
C6—C7—C8—C9	−164.56 (16)	O6A—C6—C5—C4	−150.90 (19)
C2—C7—C8—C8A	−107.0 (2)	C7—C6—C5—C4	30.1 (3)
C6—C7—C8—C8A	71.4 (2)	C3—C4—C5—C6	−52.3 (2)
C7—C8—C9—C10	−19.7 (2)	C4B—C4—C5—C6	67.8 (2)
C8A—C8—C9—C10	105.08 (19)	C4A—C4—C5—C6	−172.20 (18)
C7—C8—C9—C9A	162.58 (16)	C8C—C8B—C8A—C8F	−0.2 (3)
C8A—C8—C9—C9A	−72.7 (2)	C8C—C8B—C8A—C8	179.03 (19)
C2—C7—C6—O6A	178.46 (18)	C7—C8—C8A—C8B	55.7 (2)
C8—C7—C6—O6A	0.0 (3)	C9—C8—C8A—C8B	−68.1 (2)
C2—C7—C6—C5	−2.5 (3)	C7—C8—C8A—C8F	−125.0 (2)
C8—C7—C6—C5	179.01 (17)	C9—C8—C8A—C8F	111.2 (2)
C9A—C9—C10—N1	−174.18 (17)	C8B—C8A—C8F—C8E	1.0 (3)
C8—C9—C10—N1	8.1 (3)	C8—C8A—C8F—C8E	−178.3 (2)
C9A—C9—C10—C10A	4.3 (3)	C8A—C8B—C8C—O8C	−179.0 (2)
C8—C9—C10—C10A	−173.38 (18)	C8A—C8B—C8C—C8D	−0.2 (4)
C2—N1—C10—C9	8.7 (3)	C9A—O9A—C9B—C9C	−176.61 (19)
C2—N1—C10—C10A	−169.93 (18)	C8A—C8F—C8E—C8D	−1.3 (4)
C9B—O9A—C9A—O9B	−4.7 (3)	C8F—C8E—C8D—C8C	0.8 (4)
C9B—O9A—C9A—C9	175.31 (17)	O8C—C8C—C8D—C8E	178.7 (2)
C10—C9—C9A—O9B	−6.5 (3)	C8B—C8C—C8D—C8E	0.0 (4)
C8—C9—C9A—O9B	171.30 (19)		

### Hydrogen-bond geometry (Å, °)

**Table:**

*D*—H···*A*	*D*—H	H···*A*	*D*···*A*	*D*—H···*A*
O8C—H8C···O9B^i^	0.82	2.05	2.835 (2)	162
N1—H1···O6A^ii^	0.86	2.16	2.970 (2)	157

Symmetry codes: (i) −*x*+3/2, *y*+1/2, −*z*+3/2; (ii) *x*−1/2, −*y*+1/2, *z*−1/2.
